# Oxidatively-generated damage to DNA and proteins mediated by photosensitized UVA^☆^

**DOI:** 10.1016/j.freeradbiomed.2016.10.488

**Published:** 2017-06

**Authors:** Reto Brem, Melisa Guven, Peter Karran

**Affiliations:** The Francis Crick Institute, 1, Midland Road, London NW1 1AT, UK

**Keywords:** UVA, Photosensitizer, DNA lesions, Thiopurines, Thiopyrimidines, Protein oxidation

## Abstract

UVA accounts for about 95% of the solar ultraviolet (UV) radiation that reaches Earth and most likely contributes to human skin cancer risk. In contrast to UVB, which comprises the remaining 5% and is absorbed by DNA nucleobases to cause direct photodamage, UVA damages DNA indirectly. It does this largely through its interactions with cellular chromophores that act as photosensitisers to generate reactive oxygen species. Exogenously supplied chemicals, including some widely-prescribed medicines, may also act as photosensitisers and these drugs are associated with an increased risk of sun-related cancer. Because they amplify the effects of UVA on cells, they provide a means to investigate the mechanisms and effects of UVA-induced photodamage. Here, we describe some of the major lesions induced by two groups of UVA photosensitisers, the DNA thionucleotides and the fluoroquinolone antibiotics. In thionucleotides, replacement of the oxygen atoms of canonical nucleobases by sulfur converts them into strong UVA chromophores that can be incorporated into DNA. The fluoroquinolones are also UVA chromophores. They are not incorporated into DNA and induce a different range of DNA damages. We also draw attention to the potentially important contribution of photochemical protein damage to the cellular effects of photosensitised UVA. Proteins targeted for oxidation damage include DNA repair factors and we suggest that UVA-mediated protein damage may contribute to sunlight-induced cancer risk.

## Highlights

• Some medicines are UVA photosensitizers in patients and in cultured cells.
• Their use is associated with an increased risk of sunlight-related skin cancer.
• When combined with UVA they cause DNA damage in an oxygen-dependent manner.
• These combination induce a wide variety of DNA lesions in cultured cells.
• They also cause oxidation damage to proteins that impair cellular DNA repair capacity.

## Graphical abstract

**Figure:**
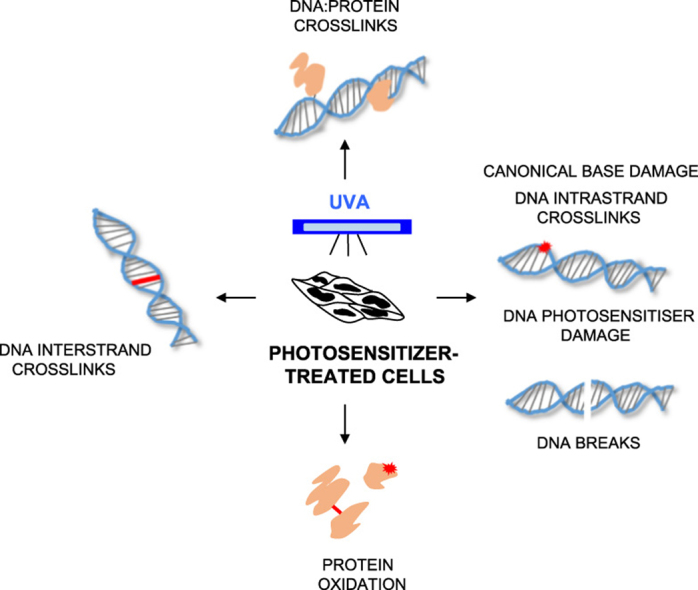

## 1 Introduction

UVA (wavelengths 320–400 nm) comprises more than 95% of the solar UV radiation that reaches Earth, making it far more abundant than UVB (280–320 nm) that accounts for the remainder. Most of UVB and all of UVC (wavelengths below 280 nm) are removed by the ozone layer and these shorter wavelengths are not present in incident sunlight. UVA is classified as “probably carcinogenic to humans” by WHO IARC [1] although, unlike UVC and UVB, it is absorbed poorly by canonical nucleotides and therefore causes much less damage to cellular DNA [2]. UVA-mediated DNA damage occurs partly by indirect mechanisms *via* interactions with cellular chromophores that act as photosensitisers to generate DNA-damaging reactive oxygen species (ROS). Depending on the distance between the chromophore and the target, UVA irradiation can also result in one-electron abstraction and the formation of a reactive radical cation. Importantly, UVA-generated ROS damage other biomolecules including proteins and lipids, and this non-DNA photodamage may be an important contributor to the biological effects of UVA such as carcinogenesis and photoaging.

Endogenous UVA chromophores have not been fully characterized, although porphyrins, flavins [3], melanin [4] and UVB photoproducts of tryptophan (6-formylindolo[3,2-*b*]carbazole, FICZ) [5] are among the potential candidates. Studies employing exogenous UVA chromophores that mimic and amplify the effects of their endogenous counterparts, provide a useful strategy to investigate events associated with UVA photosensitisers. Many of these chemicals have been used in various aspects of nucleic acid research. More importantly, some are widely-prescribed pharmaceuticals and their use is associated with an increased skin cancer risk. All these drugs have significant UVA absorbance and sensitise the formation of a variety of DNA and protein lesions. Although generally non-toxic, their foremost unifying feature is an extreme cytotoxicity in combination with low doses of UVA. This review will discuss DNA damage induced by photoactivation of thiopurines, thiopyrimidines and the fluoroquinolone group of antibiotics. We will also consider potentially important effects of photochemical damage to the proteome – particularly to DNA repair proteins.

## 2 UVA photosensitisers

**Fig. 1:**
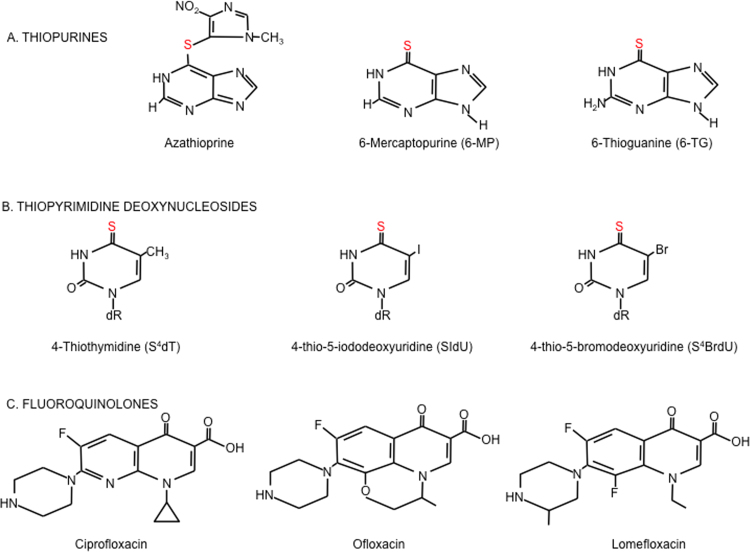
Structures of UVA photosensitisers. Azathioprine, mercaptopurine and 6-thioguanine are all converted to 6-TG deoxyribonucleotides, which are in turn incorporated into DNA. This is a prerequisite for the clinical effectiveness of thiopurines. Thiopyrimidine deoxynucleosides are incorporated into DNA of cells *via* the TK-dependent pyrimidine nucleoside salvage pathway. The fluoroquinolone class of antibiotics acts as inhibitors of DNA topoisomerases and intercalate rather than incorporate into DNA.
ii) *Thiopyrimidines*
  4-Thiothymine (S^4^T) is not currently used clinically although it has been proposed as a potential UVA photosensitiser for treatment of skin malignancies [22], [23]. Like 6-TG, S^4^T is derived from a canonical DNA base in which the replacement of a single oxygen atom by sulfur converts it to a UVA chromophore and S^4^T has an absorbance maximum at 335 nm. The S^4^T deoxyribonucleoside, 4-thiothymidine (S^4^dT) (Fig. 1B), is a good substrate for thymidine kinase (TK) and S^4^T is extensively incorporated into DNA of cells treated with S^4^dT *via* the TK-dependent pyrimidine nucleoside salvage pathway [24]. Despite its accumulation to higher levels than DNA 6-TG and the ability to undergo facile *in situ* methylation, DNA S^4^T is not detectably toxic. The absence of cytotoxicity has been ascribed to the preferential formation of structurally and thermodynamically good base pairs by both DNA S^4^T and its methylated counterpart that obviates their engagement by DNA mismatch repair, the major contributor to DNA 6-TG toxicity [25]. DNA S^4^T is, however, extremely cytotoxic in combination with low doses of UVA [24]. Recently, 2,4-dithiothymidine has been shown to be comparable or superior to 4-thiothymidine as a photosensitiser in solution and it is cytotoxic in combination with UVA [26], [27].
  The halopyrimidine nucleosides 5-iodo-2′-deoxyuridine (IdU) and 5-bromo-2′-deoxyuridine (BrdU) are also thymidine analogs. They are used extensively to label DNA where they can be detected by fluorescent antibodies or by an induced shift in DNA buoyant density. Although they retain the absorbance characteristics of the parent thymidine with maximal absorbance in the UVC range, the replacement of the methyl group with I or Br alters their reactivity. DNA IdU and BrdU are photoactive and synergistically enhance the toxicity of UVC and UVB [28], [29]. When activated by short wavelength UV, DNA-embedded IdU or BrdU induce potentially lethal DNA lesions such as strand breaks [30], DNA interstrand crosslinks (ICLs) [31] and DNA protein crosslinks (DPCs) [32]. DNA halopyrimidines also sensitise cells to UVA wavelengths when they are combined with the DNA-intercalating Hoechst dye that serves as a surrogate UVA chromophore [33]. The synergistic cytotoxicity of halopyrimidine/Hoechst/UVA combinations led to the development of thio analogs of IdU and BrdU, 5-iodo-4-thio-2′-deoxyuridine (SIdU) and 5-bromo-4-thio-2′-deoxyuridine (SBrdU) [34], [35] (Fig. 1B). As anticipated, SIdU and SBrdU are UVA chromophores with an absorbance maximum at around 340 nm. They are salvaged reasonably well for incorporation into DNA and are minimally toxic. When combined with UVA, however, DNA-embedded halothiopyrimidines induce a spectrum of potentially lethal DNA damage that does not require the participation of Hoechst dye [36].
iii) *Fluoroquinolones*
  The fluoroquinolones including ciprofloxacin, ofloxacin and lomefloxacin (Fig. 1C) are among the most extensively prescribed antibiotic drugs worldwide. Fluoroquinolones are acknowledged photosensitisers [37] with both UVA and UVB absorbance maxima. In combination with UVA, they are photocarcinogens in mice [38], [39] and are associated with adverse skin reactions and an increase of pre-malignant skin lesions in patients [40], [41], [42], [43], [44]. The bactericidal effect of the fluoroquinolones reflects inhibition of DNA gyrase and topoisomerase IV [45]. They are not toxic to human cells although they are synergistically cytotoxic when combined with low doses of UVA.

i) *Thiopurines*
  The thiopurines azathioprine, mercaptopurine and 6-thioguanine (6-TG) (Fig. 1A) are effective anticancer, anti-inflammatory and immunosuppressant drugs (reviewed in [6]). They are all prodrugs that undergo enzymatic conversion that culminates in the formation of the thiopurine nucleotides that are an absolute requirement for their clinical effectiveness. Despite more than half a century of clinical use, the molecular events underlying thiopurine cytotoxicity are still not fully understood. Suggested mechanisms include inhibition of *de novo* purine synthesis resulting in an inadequate supply of purine nucleotides for replication and transcription [7] and interference with intracellular signalling pathways *via* competition for GTP binding by G proteins [8], [9]. Thioguanine nucleotides are substrates for incorporation into DNA and to a lesser degree into RNA and the biological effects of thiopurines are at least partly dependent on the formation of DNA 6-TG [7], [10]. DNA 6-TG may undergo *in situ* non-enzymatic methylation that can provoke ultimately lethal processing by DNA mismatch repair [11], [12]. Alternatively, it can participate in the formation of DNA interstrand-crosslinks [13] that are highly toxic in a mismatch repair-independent manner. The methylated form of DNA 6-TG miscodes during replication and it is noteworthy that azathioprine treatment is associated with a perceptible increase in mutation frequency in circulating lymphocytes [14] and with an increased risk of leukemia [12]. Most striking, however, is the greater than 100-fold higher risk of skin cancer in immunosuppressed organ transplant patients [15], most of whom will have been prescribed azathioprine and whose skin contains detectable amounts of DNA 6-TG [16]. Its more intermittent use in the management of inflammatory bowel disease entails a lower, but still significant skin cancer risk [17], [18], [19]. Sunlight exposure is a contributory factor in thiopurine-related skin cancer. The skin of patients taking azathioprine is photosensitive to UVA but not to UVB, consistent with the absorbance maximum of DNA 6-TG at around 340 nm. This has led to the suggestion that the photochemical reactions of azathioprine or its metabolites [20] may contribute to skin cancer risk [21].

## 3 UVA photosensitisation

UVA photosensitisation occurs by two main mechanisms that can trigger one-electron oxidation of suitable substrates (Type I) and/or generate various reactive oxygen species (ROS) including superoxide (O_2_^•-^, by Type I) or singlet oxygen (^1^O_2_) (Type II) [46], [47]. Following UVA excitation of the chromophore, Type I reactions generate a pair of charged radicals (a photosensitiser anion and a target cation). Both radicals can undergo further reactions to produce oxygenated products. In the case of guanine, for example, the related cation may undergo hydration reactions which gives rise to the 8-hydroxy-7,8-dihydroguanyl radical which upon one-electron oxidation mediated by molecular oxygen results in the formation of 8-oxo-7,8-dihydroguanine (8-oxoGua). Alternatively, the photosensitiser anion may be oxidised back by O_2_ in a reaction which generates O_2_^•-^. Enzymatic dismutation of O_2_^•-^ generates H_2_O_2_ which although relatively unreactive, is freely diffusible throughout the cell and can generate highly destructive hydroxyl radicals (^•^OH) *via* metal catalysed reactions. In Type II reactions, the energy absorbed by the chromophore is transferred directly to molecular oxygen to generate ^1^O_2_, a relatively long-lived ROS. In canonical DNA, ^1^O_2_ targets exclusively DNA guanine to generate 8-oxoGua [48], [49].

## 4 Nucleobase photoproducts

**Fig. 2:**
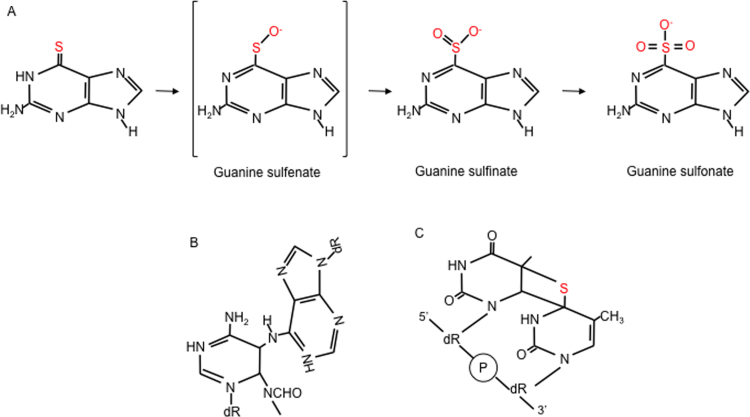
UVA photoproducts of DNA 6-thioguanine (6-TG) and 4-thiothymidine A. 6-TG. Reactive oxygen species generated from the interaction between 6-TG and UVA oxidise 6-TG or DNA 6-TG to guanine sulfinate and sulfonate as predominant products. These oxidised forms are also produced by MMPP treatment of 6-TG-substituted DNA. B.Potential intrastrand crosslink between DNA 6-TG and an adjacent imidazole ring-opened deoxyadenosine. UVA-mediated generation of covalent adducts of this type between 6-thioinosine and deoxyadenosine has been demonstrated in solution. C.4-thiothymidine. The thietane photoproduct generated by interstrand crosslinking between DNA 4-thiothymidine and a 5’-DNA thymidine.
  The formation of UVA-mediated intrastrand crosslinks between DNA 6-TG and neighbouring nucleotides has not been demonstrated. The generation of DNA 6-TG:A addition products is plausible based on the known photoaddition reaction between 6-TG and 2’-deoxyadenosine containing an opened imidazole ring in solution [59], [60], although this reaction might be disfavoured in DNA by steric constraints (Fig. 2B).
  Oxidised forms of 6-TG are bulky lesions and, like intrastrand crosslinks, they distort and destabilize the DNA helix. This property makes them potential targets for removal by nucleotide excision repair (NER) that rids DNA of this type of damage. DNA GSO_3_ is not actively removed, however, and NER-deficient cells are not hypersensitive to treatment with 6-TG+UVA [10], [16]. These findings suggest that 6-TG+UVA does not generate potentially lethal NER substrates. It should be noted, however, that NER is impaired by the protein oxidation induced by 6-TG+UVA [61]. This photochemical protein damage provides an alternative explanation for the persistence of DNA GSO_3_ and the absence of sensitivity of NER-deficient cells.
ii) *Thiothymidine*
  When S^4^dT is UVA irradiated in dilute aqueous solution, the major reaction is hydrolysis to dT. Small amounts of thymidine sulfenate (TSO) and a dimeric form, tentatively identified as T-S-T, are also produced. dT is not a significant photoproduct of DNA S^4^dT, however, and it is not detected in irradiated double-stranded oligonucleotides [62]. Degradation of DNA-embedded S^4^T is heavily dependent on sequence context. It is atypically photosensitive when placed 3’ to thymine. The reason for this selective photosensitivity is the preferential formation of DNA intrastrand crosslinks between 5’-T and 3’-S^4^T. Based on its behaviour on RP-HPLC, fluorescence spectrum and recognition by specific antibodies, the crosslinked species was identified as a thietane, S^5^-(6-4 TT), the thio analog of the related oxetane pyrimidine (6-4)-pyrimidone (6-4 TT), a DNA photoproduct of both UVC and UVB (Fig. 2C). Unlike the UVC-induced oxetane which rapidly converts into a ring-open form, the thietane is more stable and the ring-closed structure is favoured. Thietanes were detected in human cells treated with S^4^dT /UVA and they were repaired by NER, albeit somewhat more slowly than canonical pyrimidine (6-4)-pyrimidone (6-4 PP) photoproducts. DNA thietanes are therefore likely to be significant contributors to the particular S^4^dT/UVA sensitivity of NER-deficient XPA cells [24], [62]. In contrast to 6-TG, ROS do not appear to contribute significantly to the phototoxicity of S^4^dT at low UVA doses [62]. Paradoxically, however, UVA irradiation of S^4^dT in solution yielded similar (or higher) ^1^O_2_ yields to irradiated 6-TG [26], [63].
iii) *Halothiopyrimidines*
  UVA degrades SIdU both in free solution and in DNA. An initial UVA-induced deiodination gives rise to a thiouracil-5-yl radical that can be further converted to SdU. Higher UVA doses generate 2’-deoxyuridine (dU). dU formation most likely proceeds *via* an oxidised thiol intermediate as treatment of authentic SdU with MMPP also generates dU and an intermediate with the same properties is detectable after irradiation of either SIdU or SdU (Fig. 3). In DNA, thiouracil-5-yl radicals derived from SIdU can react further to generate DNA strand-breaks, ICLs or DPCs (see below). Irradiation of cells containing DNA SIdU also produces a non-toxic DNA lesion, most likely uracil, that is a substrate for the uracil DNA glycosylase (UNG) [36].

i) *6-Thioguanine*
  The mechanism by which the ultimate thiopurine metabolite, DNA 6-TG, exerts its photochemical effects, is still a matter of debate. Several studies have implicated ^1^O_2_ generated by a Type II photosensitisation in photochemical DNA damage mediated by 6-TG [50], [51], [52], [53]. A recent study [54] which revealed a much lower ^1^O_2_ yield than previously reported, questions the dominance of ^1^O_2_ in 6-TG-mediated photo-oxidation. DNA 6-TG itself is a major target of photochemically generated ROS because its oxidation potential is lower than that of canonical DNA bases. Guanine-6-sulfinate (GSO_2_) and guanine-6-sulfonate (GSO_3_) (Fig. 2A) were originally identified as oxidation products of 6-TG in free solution [55]. Complete UVA-mediated degradation of 6-TG in aqueous solution yields GSO_2_ as the major photoproduct (60%) and minor amounts of GSO_3_, guanine and guanine-6-thioguanine (a 6-TG addition product) [56]. These reactions are dependent on molecular oxygen and involve Type II photosensitisation [51], [52]. DNA GSO_2_ and GSO_3_ are also the major products when double-stranded DNA containing 6-TG is UVA irradiated or treated with magnesium monoperoxyphthalate (MMPP), a mild oxidising agent that is able to oxidise DNA 6-TG but not the canonical DNA bases [52]. GSO_2_ and GSO_3_ comprise more than 90% of identified DNA 6-TG photoproducts at low and medium UVA doses. Increasing UVA doses result in higher GSO_3_ recovery, concomitant with decreased GSO_2_ levels [56]. The photochemistry of DNA 6-TG is thus consistent with sequential oxidation to DNA GSO_3_ *via* guanine sulfenate (GSO) and GSO_2_. GSO_3_ cannot be oxidised further [50], [57]. A postulated GSO intermediate is not observed. This may reflect its instability although the possible formation of GSO_2_ and GSO_3_ *via* an initial peroxy intermediate [58] has not been excluded.

**Fig. 3:**
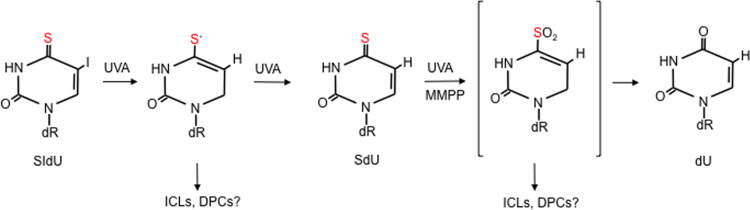
UVA photoproducts of 4-thio-5-iododeoxyuridine (SIdU). UVA irradiation of free SIdU in solution or of DNA containing incorporated SIdU causes deiodination and the generation of SdU *via* a reactive a 5-thiyl radical. Further reactions induced by UVA or MMPP generate (unidentified) thiol-oxidised intermediates – possibly the sulfinate [bracketed]. The intermediate(s) can undergo loss of the thiol group to generate dU as a final product. The thiyl radical and the oxidised thiol DNA intermediates can also react with nucleobases on the complementary DNA strand to generate DNA interstrand crosslinks (ICLs) or potentially with protein functional groups to form DNA-protein crosslinks (DPCs).
  Consistent with the relative stability of the C5-Br bond, UVA irradiation of SBrdU does not induce significant debromination. SBrdU in solution is nevertheless completely degraded by UVA and small amounts of BrdU are formed. UVA-mediated degradation of SBrdU coincides with the formation of an ephemeral intermediate that disappears concomitantly with BrdU formation (Fig. 4). As with SdU, MMPP treatment mimics the effects of UVA and is consistent with BrdU formation *via* S4 oxidation and loss of the oxidised thiol group. The effect of UVA on DNA-embedded SBrdU is very different. Approximately 50% of DNA SBrdU is degraded by very low UVA doses whereas the remainder is much more refractory even at high doses. Whether this differential sensitivity to degradation reflects the effects of particular sequence contexts is currently unclear. BrdU formation is not observed, possibly due to preferential reactions between the oxidised thiol group and other DNA nucleobases or proteins (to form ICLs and DPCs, respectively). UNG-deficient cells are sensitive to SBrdU/UVA, indicating that this combination also induces unidentified potentially lethal DNA lesions that are substrates for excision by UNG [36].

**Fig. 4:**
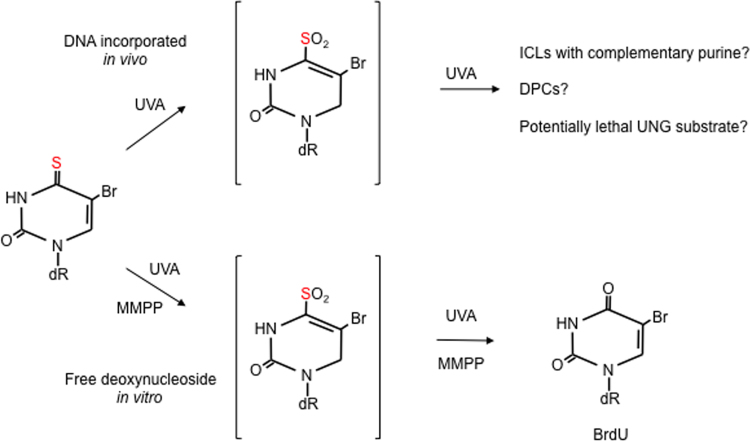
UVA photoproducts of 4-thio-5-bromodeoxyuridine (SBrdU). In free solution, UVA irradiation or MMPP treatment of the deoxynucleoside generates 5-bromodeoxyuridine (BrdU) *via* an (unidentified) oxidised intermediate (shown here as the sulfinate). The photochemistry of DNA SBrdU is different. UVA-mediated degradation of SBrdU is not followed by the formation of detectable BrdU. This suggests that reaction with the complementary DNA strand or proteins in preferred. A potentially lethal DNA lesion that is a substrate for the *Escherichia coli* uracil-DNA N-glycosylase (UNG) has not been identified. UVA does not induce debromination of SBrdU either in free solution or in DNA.
  NER defective cells are hypersensitive to both SBrdU/UVA and SIdU/UVA. This indicates that these combinations induce potentially lethal DNA photolesions that most likely resemble S^5^-(6-4)TT and the canonical UV (6-4)PP photoproducts [36].
iv) *Fluoroquinolones*
  Fluoroquinolones do not become covalently incorporated into DNA. They can, however, enhance the formation of canonical DNA lesions. UVC and UVB induce several different DNA cyclobutane pyrimidine dimers (CPDs) by direct photon absorbance. UVA generates exclusively thymine<>thymine (T<>T) CPDs most likely predominantly by triplet energy transfer from excited cellular chromophores to DNA thymine [47]. Some (examples include lomefloxacin and ciprofloxacin), but not all (ofloxacin and rufloxacin) UVA-excited fluoroquinolones have sufficiently high triplet energy levels to mediate T<>T formation by an analogous triplet energy transfer [64], [65]. (6-4)PP photoproducts are not formed by these mechanisms.

## 5 DNA interstrand crosslinks (ICLs)

ICLs are highly cytotoxic lesions. In combination with UVA, 6-TG, S^4^T and the halothiopyrimidines all induce ICLs in duplex oligonucleotides *in vitro* [36], [62], [66], indicating that thionucleotides have the potential to form ICLs *in vivo*.

ICLs in living cells are processed by the Fanconi Anemia (FA) pathway, homologous recombination, NER and translesion DNA synthesis which together effect their repair. FA cells are hypersensitive to crosslinking agents and their response is considered diagnostic for ICL induction since an active FA pathway is essential for ICL repair. Monoubiquitination of the FANCD2 protein is a key step in FA pathway activation and provides a sensitive indicator of the presence of ICLs. UVA irradiation of cells treated with 6-TG, S^4^dT or the halothiopyrimidine deoxynucleosides induces monoubiquitination of FANCD2 and FA cells are hypersensitive to all these drug/UVA combinations [13], [36], [62].

i) *6-Thioguanine*
  UVA induces efficient crosslinking of double-stranded oligonucleotides heavily substituted with 6-TG [66]. In these model substrates, most ICLs are formed *via* disulfide bridges and crosslinking is significantly enhanced by closely opposed 6-TGs. A minority of ICLs involve 6-TG linkage to a normal base *via* a single sulfur atom. These ICLs most likely predominate in UVA-irradiated cells with sparsely distributed DNA 6-TG. Their formation may involve generation of a thiyl radical in a Type I photosensitisation followed by reactions with nucleobase amino groups.
  In cells, 6-TG induces ICLs even without UVA treatment. FA cells are hypersensitive to 6-TG concentrations that induce FANCD2 ubiquitination in wild-type cells [66], [67]. Treatment with 6-TG depletes cellular antioxidant defences and cause an increase in steady-state levels of ROS. ROS induce ICLs by targeting DNA-embedded 6-TG [13]. Because antioxidant depletion is expected to increase steady-state O_2_^•-^ and H_2_O_2_ levels and subsequently to generate ^•^OH, it is likely that 6-TG-induced ICLs may be generated *via* the formation of thiyl radicals, but the crosslinking mechanism has not been elucidated. The intense ROS burst that accompanies UVA irradiation further increases ICL formation. 6-TG- and 6-TG/UVA-induced ICLs are easily detectable by physicochemical methods such as the comet assay. Their formation can also be inferred from the chromosome aberrations present in the karyotypes of treated cells. The observed radial chromosomes and chromosome breaks are characteristically associated with aberrantly resolved ICLs [66].
ii) *Thiothymidine*
  UVA also crosslinks duplex oligonucletides containing S^4^T. ICL formation is largely independent of sequence context although it is less efficient when S^4^T has a flanking T, most likely because of the preferential formation of intrastrand photoproducts [62]. UVA-induced crosslinking is between S^4^T and the complementary A. It is abolished when the A is replaced by G and diminished when the opposed base is inosine or 2-aminopurine.
  Treatment with S^4^dT/UVA (but not S^4^dT alone) also induces ICLs *in vivo*. Low UVA doses (≤10 kJ/m^2^) induce FANCD2 ubiquitination and ICL formation in cells containing DNA-incorporated S^4^T. Consistent with ICL induction, FA cells and cells defective in XPF, another essential ICL processing factor, are exquisitely sensitive to DNA S^4^T/UVA [62]. S^4^dT /UVA-treated FA cells also accumulate the characteristic radial chromosomes and chromosome breaks associated with imperfectly processed ICLs (E. McAdam, P. Karran, unpublished).
  Phosphoramidites of SIdU or SBrdU are not available and this has precluded examination of crosslinking in synthetic halothiobase-containing oligonucleotides. However, UVA irradiation of a double-stranded oligonucleotide containing a single SdU, a photoproduct of SIdU, does induce crosslinking to a complementary A or inosine. This observation indicates that ICL formation is favoured by UVA-induced SIdU deiodination. Consistent with ICL formation, FA cells are hypersensitive to both SBrdU/UVA and SIdU/UVA and both treatments trigger FANCD2 ubiquitination [36]. ICL formation by these combinations is enhanced in D_2_O, indicating that it is at least partially by Type II photosensitisation [36]. ICL formation may also involve the highly reactive thiyl or 5-uracily radicals (Fig. 3).

## 6 Collateral damage

### 6.1 DNA

With the lowest oxidation potential among canonical DNA nucleobases, guanine is the most susceptible to damage by ROS. DNA 8-oxoGua, the predominant product of ^1^O_2_-mediated DNA oxidation, is present in cultured cells treated with 6-TG/UVA or azathioprine/UVA. Approximately half of the 8-oxoGua is generated from DNA-incorporated 6-TG. The remainder is the product of ROS derived from the pool of unincorporated 6-TG nucleotides [20]. The potential importance of 6-TG-mediated guanine oxidation is underlined by the observation that increased urinary 8-oxodG levels are associated with skin cancer risk in transplant patients taking azathioprine [68]. It is noteworthy in this regard that the estimated oxidation potential of 6-TG is equal to or lower than that of 2’-deoxyguanosine, suggesting that it may effectively act as a sink for oxidatively-generated damage in DNA 6-TG [54].

In contrast, treatment of cultured cells with combinations of low dose (≤10 kJ/m^2^) UVA and thiopyrimidines does not generate ROS - at least those detectable by the rather limited CM-H_2_DCFDA FACS assay - and DNA S^4^T/UVA [62], and SIdU/UVA or SBrdU/UVA combinations [36] are not associated with measurable increases in DNA 8-oxoGua despite extensive toxicity.

UVA activated fluoroquinolones are a source of ROS. They damage DNA and induce oxidised pyrimidine and purine bases including 8-oxoGua as a major photoproduct [69], [70], [71]. DNA 8-oxoGua levels vary depending on the fluoroquinolone [71]. In isolated DNA, UVA-activated fluoroquinolones generate ^1^O_2_ and DNA 8-oxoGua in a Type II photoreaction. In yeast, the combination of rufloxacin (which has a very low triplet energy and cannot induce T<>T CPDs) and UVA induces predominantly G to T transversions, the characteristic mutation associated with DNA 8-oxoGua. Confirming the involvement of DNA 8-oxoGua, *ogg-1*^-/-^ strains in which the repair of these lesions is defective, are hypersensitive to mutation by rufloxacin/UVA [72]. In general, however, triplet energy transfer to generate T<>T CPDs appears to be the predominant mode of fluoroquinolone-induced DNA damage [71].

It is also worth mentioning that like many antibiotics, the fluoroquinolones (so far demonstrated for norfloxacin and molifloxacin) induce oxidative stress that contributes to their bactericidal activity [73]. This common oxidation-related toxicity occurs at least partly as a consequence of oxidation of the guanine nucleotide pool and the incorporation of 8-oxoGua into nucleic acids [74].

### 6.2 Protein

Cellular proteins are significant targets for damage by ROS [75]. Oxidised proteins are relatively insoluble and the intracellular deposition of insoluble oxidised protein aggregates is associated with aging and with several neurodegenerative and inflammatory disorders. Largely overlooked by the DNA repair field in the past, protein oxidation is a significant contributor to radiation-induced toxicity and mutagenesis [76] and there is growing evidence that excessive ROS can damage the human DNA repair proteome and compromise DNA repair [61], [65], [77].

Amino acid side chains can be oxidised to generate protein carbonyls (aldehydes and ketones) as stable products [78]. The sulfur atoms of methionine and cysteine can be oxidised to the corresponding sulfenic acids (-SOH), unstable products that either undergo disulfide bond formation or further oxidation to sulfinic (-SO_2_) or sulfonic acid (-SO_3_) derivatives [79]. ROS generated by UVA radiation cause extensive protein oxidation that is significantly amplified by exogenous photosensitisers. 6-TG, fluoroquinolones, riboflavin and FICZ all increase susceptibility to UVA-induced protein carbonylation and thiol oxidation by reactions that are at least partly ^1^O_2_ dependent [61], [65], [77] (R.Brem, unpublished). Among the protein changes induced by these treatments, crosslinking between the components of multisubunit DNA repair complexes is particularly noteworthy. Photosensitiser-dependent intersubunit crosslinking has been observed for the PCNA [80], Ku [61], RPA [81] and MCM2-7 [77] DNA replication/repair complexes.

## 7 DNA-protein crosslinks

UVA activation of DNA 6-TG also induces DPCs. *In vitro* experiments demonstrated the slow formation of DPCs between 6-TG-containing oligonucleotides and thiol or amino groups in oligopeptides [82]. Crosslinking was dependent on 6-TG oxidation. It was induced by UVA irradiation of the oligonucleotide and was optimal following oxidation by MMPP, which is consistent with the involvement of GSO_3_, a good leaving group in nucleophilic substitution reactions. Low-dose UVA irradiation also induced extensive DNA-protein crosslinking in cells containing DNA 6-TG [82]. DPC formation was rapid suggesting that the mechanism differs from that of *in vitro* crosslinking. 2-D DIGE analysis identified the DNA repair/replication proteins MSH2, PCNA, DDB-1 and MCM2 among the crosslinked species.

DNA-incorporated 6-TG is an important contributor to UVA-induced DPC formation in intact cells. DNA 6-TG serves as both a target for crosslink formation and as a source of ROS. Confirmation that ROS also induce crosslinking between proteins and canonical DNA nucleobases was obtained from a comparative proteomic analysis of DPCs formed in cultured human cells treated with 6-TG or (the non DNA-integrated) ciprofloxacin in combination with UVA [83]. Stable isotope labelling with amino acids in cell culture (SILAC) and mass spectrometry identified more than 2000 DNA-crosslinked proteins most of which are involved in gene expression or DNA repair/replication. Essential proteins in all of the known canonical DNA repair pathways were represented among those crosslinked by 6-TG or ciprofloxacin combined with UVA. Among these DNA repair proteins, more than 75% were present in DPCs induced by both 6-TG+UVA and ciprofloxacin+UVA (Table 1).

**Table 1:** DNA repair proteins most vulnerable to crosslinking by 6-TG+UVA and ciprofloxacin+UVA.

**Protein**	**6-Thioguanine+UVA**	**Ciprofloxacin+UVA**
**APEX (APE1)**	**+**	**+**
**BLM**	**+**	**+**
**DDB1**	**+**	**+**
**DUT**	**+**	**+**
**FEN1 (DNase IV)**	**+**	**+**
**LIG1**	**+**	**+**
**LIG3**	**+**	**+**
**MRE11A**	**+**	**+**
**MSH2**	**+**	**+**
**MSH6**	**+**	**+**
**PARP1 (ADPRT)**	**+**	**+**
**PCNA**	**+**	**+**
**POLD1**	**+**	**+**
**POLD3**	**+**	**+**
**POLE3**	**+**	**+**
**PRF19 (PSO4)**	**+**	**+**
**RAD50**	**+**	**+**
**REQL (REQ1)**	**+**	**+**
**RPA1**	**+**	**+**
**XRCC1**	**+**	**+**
**XRCC5 (Ku80)**	**+**	**+**
**XRCC6 (Ku70)**	**+**	**+**
**MGMT**	**+**	–
**MMS19**	**+**	–
**MPG**	**+**	–
**NBN (NBS1)**	**+**	–
**NUDT (MTH1)**	**+**	–
**POLE1**	**+**	–
**PRKDC**	**+**	–
**UBE2N (UBC13)**	**+**	–
**CHAF1A (CAF1)**	–	**+**
**FANCD2**	–	**+**
**FANCI**	–	**+**
**MSH3**	–	**+**
**TP53**	–	**+**
**TP53BP1 (53BP1)**	–	**+**
**WRN**	–	**+**

UVA irradiation of cells containing DNA SIdU or SBrdU also generates DPCs [36]. Their formation and the crosslinked proteins have not, however, been analysed in detail.

## 8 DNA damage, repair and cancer

### 8.1 Mutagenesis

One consequence of exposure to clinical photosensitisers and sunlight is likely to be the continuous production of potentially mutagenic DNA lesions. The chronic induction of DNA damage effectively mimics inefficient DNA repair. The correlation between ineffective DNA repair and cancer risk is exemplified by the cancer proneness of patients with xeroderma pigmentosum (defective NER), DNA breakage syndromes (deficiencies in repair factors such as DNA ligase IV, NBS or ATM), Fanconi Anemia, Lynch Syndrome (DNA mismatch repair-deficient) and MUTYH-associated polyposis (impaired DNA 8-oxoGua processing).

Damage to the proteome also compromises DNA repair efficiency and photochemical oxidation of the PCNA, Ku, hOGG1, MUTYH and RPA DNA repair proteins in cultured human cells is associated with the inhibition of their respective DNA repair pathways [61], [65], [77], [80], [81].

The UVA photosensitisers we have considered here mimic and amplify the effects of their endogenous cellular counterparts. Photosensitised UVA radiation and oxidative stress in general oxidises DNA repair proteins and compromises DNA repair in human cells. UVA is firmly implicated in the photosensitivity of, and the hugely increased incidence of skin cancer in patients taking azathioprine [21], [84] and it is likely that inhibition of NER caused by the interaction between DNA 6-TG and UVA is a contributory factor. It is significant in this regard that sequencing studies indicate that skin tumors [85], [86], [87], [88] and even normal sun-exposed skin [89] accumulate very high numbers of mutations that bear the signature of UVB-induced DNA lesions that are substrates for removal by NER. These unexpectedly high mutational burdens suggest that chronic, low-level protein damage caused by the UVA component of solar radiation may reduce the efficiency of NER and contribute to the development of skin cancer.

### 8.2 Chemotherapy

The introduction of potentially lethal DNA lesions remains a mainstay of chemotherapy. The synergistic lethality of UVA and the thiopyrimidines has led to suggestions that S^4^dT, SIdU or SBrdU treatment combined with low dose UVA might represent a useful photochemotherapeutic strategy [22], [23]. Indeed, a preliminary study of the effects of combined intravenous S^4^dT and fiber optic UVA in an orthotopic rat bladder tumor model indicated that this might represent a viable therapeutic option [90]. Recent studies indicate that 2,4-dithiothymine may offer an improved phototherapeutic option because of its longer activation wavelength (affording greater tissue penetration) and higher triplet and ^1^O_2_ yields [26], [27]. These properties are shared by 2,4-dithiouracil and this has led to the recent suggestion that it may represent a potential RNA-targeted phototherapeutic agent [91].

DNA repair efficiency is an important determinant of tumor responsiveness. The excellent response of testicular tumors to cisplatin is partly due to their relatively inefficient NER [92]. Similarly, the favourable response of BRCA1-mutated breast carcinomas to poly(ADP-ribose) polymerase (PARP) inhibitors reflects the amplified toxicity of DNA breaks that accumulate owing to defective recombinational DNA repair [93]. Tumor cells are frequently in a state of oxidative stress and this may contribute to a favourable response to chemotherapy. The toxicity of platinum drugs, for example, is enhanced by the increased oxidative stress induced by parenteral ascorbate treatment [94] or by depletion of cellular NADPH [95], the main cellular reducing agent. Indeed, carboplatin-based chemotherapy combined with simultaneous oxidative stress results in improved responses in ovarian cancer patients [94]. Photochemotherapeutic approaches that ally induced oxidative stress to the induction of potentially toxic DNA lesions would represent a two-pronged attack on malignancies: induction of DNA damage and inhibition of repair.

In summary, it seems necessary to take a holistic view when analysing the biological effects of photosensitisers. In addition to the obvious importance of DNA lesion induction, the possible amplifying effect of damage to the DNA repair proteome that normally avert the consequences of those lesions should also be considered.
